# Perceptions regarding the use of cannabis in orthopaedics for treating musculoskeletal joint pain: A survey of arthritis patients

**DOI:** 10.1177/03000605261438343

**Published:** 2026-05-06

**Authors:** Marko Gjorgjievski, Kim Madden, Daniel Tushinski, Anthony Adili, Conner Bullen, Frank Koziarz, Alex Koziarz, Lauren de Freitas, Silvia Li, Mohit Bhandari, Herman Johal

**Affiliations:** 1 Division of Orthopaedic Surgery, Department of Surgery, Queen’s University, Canada; 2 Department of Health Research Methods, 3710 Evidence and Impact, McMaster University, Canada; 3 Division of Orthopaedic Surgery, McMaster University, Canada; 4 Royal College of Surgeons Ireland, Ireland; 5 Department of Medical Imaging, 3710 McMaster University, Canada; 6 University of Toronto Institute of Medical Science, Canada; 7 Department of Surgery, University of Calgary, Canada

**Keywords:** Cannabis, analgesics, orthopedic surgery, postoperative pain, pain management

## Abstract

There is renewed interest in investigating the analgesic properties of cannabis for musculoskeletal joint pain; however, available data remain limited. This cross-sectional study was designed with the objective of evaluating patients’ perceptions regarding the effect of cannabis on arthritis pain. Patients with arthritis pain presenting at one of three orthopedic clinics were asked to complete the study questionnaire. Outcomes were the perceived effect of cannabis on arthritic pain (measured using the continuous visual analog scale, with scores ranging from 0% to 100%) and association between preferences, attitudes, and barriers to the clinical use of cannabis for arthritic pain (evaluated using multivariable linear regression analyses). Sample size was also calculated using multivariable regression analysis. The study included 406 patients, including 105 (26.3%) who had already previously used cannabis for medical purposes and 63 (15.8%) who had used it during the past year. Approximately one-third of the patients who were prescribed opioids (78/256, 30.5%) had used them in the last week. On an average, patients stated that cannabis could treat 53.6% ± 2.6% of their pain (95% confidence interval = 51.1%–56.1%) and helped them replace 50.4% ± 3.2% of their analgesic medications (95% confidence interval = 47.2%–53.6%). Additionally, 88.8% of the patients (135/152) believed that cannabis would aid pain management. Individuals who had used cannabis for medical purposes (odds ratio = 7.2, 95% confidence interval = 1.6–12.8, *p* = 0.001) and patients reporting more severe baseline pain (β = 0.2 per point, 95% confidence interval = 0.1–0.3, *p* = 0.012) were more likely to report meaningful pain improvement. A quarter of the patients with arthritis already used cannabis, and many patients regarded cannabis as an effective pain treatment option. Patient preferences regarding administration and distribution, timing, and indications may help inform clinicians regarding the optimal utilization of cannabis in patients with joint and arthritis pain.

## Introduction

Approximately 600 million people in the world have arthritis,^1,2^ with one in every four individuals reporting severe joint pain. It is among the top causes of overall disability and reduced quality-of-life,^3,4^ causing limitations in daily activities in approximately 24 million adults.^1^ Annual medical care costs and lost earnings due to arthritis are also estimated to be over US$300 billion.^1^

Pain control following surgery is crucial as postoperative pain can limit early mobilization.^5,6^ One of the main treatments for patients with severe symptomatic osteoarthritis (OA) and significant quality-of-life limitations is total joint arthroplasty (TJA).^7^ Opioids are among the most commonly used pain medications for postoperative pain control,^8–10^ with almost 8% of all opioid prescriptions in the USA coming from orthopedics.^11,12^ Considering that 20% of opioid-related deaths involve prescription medications,^13,14^ opioid prescribing has undoubtedly contributed to the current opioid crisis, particularly in Canada, the USA, the UK, and Australia.^15–19^ In the USA, >90 opioid-related deaths are reported every day, and in Canada, opioid-related hospitalizations and emergency department visits have risen by more than 50% over the last decade, with the highest rise in the past 3 years.^20–22^

Postoperative opioid overprescription seems to be a problem unique to North America as patients in other parts of the world consume opioids less frequently and in lower doses.^23–25^ The USA is responsible for the consumption of 80% of the global opioids supply.^26^ Opioid prescriptions showed a decline between 2012 and 2017; however, the number of days per prescription continued to rise.^27,28^ This indicates that orthopedic surgeons in Canada and the USA still regard opioids as the primary mode of postoperative pain control; therefore, exploring nonopioid analgesic alternatives will serve a critical role in curbing this problem.

Although previously cannabis was often regarded as a substance with no medical benefits and high abuse potential, over the past several years, perceptions regarding its utilization have undergone a significant transformation, allowing a more permissive approach. Cannabis modulates pain primarily through the endocannabinoid system (ECS) and activation of cannabinoid receptors (CB1 and CB2), which inhibits painful signals and reduce inflammation as well as through interactions with noncannabinoid targets, with studies supporting the analgesic properties of cannabis.^29^ A living systematic review examined data primarily from randomized controlled trials (RCTs) and evaluated the benefits and harms of cannabinoids (including cannabis-related products) in adults with chronic pain; this review reported reduction in pain severity, particularly in case of neuropathic pain, compared with placebo.^30^ However, there is lack of robust data on the use of cannabis for pain management in orthopedic surgery, highlighting the need for further investigation.^26,31^ Considering that cannabis use is completely legal in Canada, investigating its potential use for arthritic pain presents a distinct research opportunity.^32^

The goals of this survey were to examine patients’ perceptions regarding cannabis use for treating arthritis pain. Evaluation of the perceived effects of cannabis on joint and arthritis pain was the primary objective, while exploration of patients’ views and preferences surrounding cannabis use in the orthopedic setting was the secondary objective. We hypothesized that patients would perceive cannabis to be an effective option for managing arthritis-related pain.

## Methods

This cross-sectional study used a survey-based approach to determine orthopedic clinic patients’ perceptions, preferences, and insights regarding cannabis use for musculoskeletal (MSK) joint pain. Patients who presented at three participating orthopedic clinics in Canada from 24 January 2018 to 7 March 2018 were screened for inclusion. Eligibility criteria included English-speaking patients aged >18 years presenting with arthritis or joint pain. Cognitively impaired patients or those who were too ill or injured to participate were excluded. Our center also conducted separate studies using similar methods, examining patients with traumatic injury or back pain and their experiences with cannabis.^33,34^ All the included patients provided informed consent before study enrollment. The relevant Research Ethics Board provided approval for the study. This was a level of evidence III study.

We used a modified version of a questionnaire used in previous studies, which was created using inputs from existing research studies, patients, and a group of specialists, including orthopedic surgeons, anesthesiologists, rehabilitation professionals, and epidemiologists.^33,34^ The questionnaire included 50 questions (multiple-choice or Likert-scale format)^35^ regarding the following: (a) patient and injury characteristics; (b) pain severity and analgesics usage; (c) perceptions and positions concerning the medical use of cannabis; (d) perceived effectiveness of cannabis use for arthritis and joint pain; and (e) barriers to appropriate clinical use. We assessed pain severity using the continual visual analog scale (VAS), with scores ranging from 0 (for no pain) to 100 (indicating unbearable pain). A continuous scale ranging from 0% to 100% was used to measure the perceived effectiveness of cannabis use by asking patients to rate the level of pain they believed was being (or could be) treated using cannabis; this served as the primary outcome of the study. Secondary outcomes included preferences, attitudes, and barriers to the clinical use of cannabis for joint pain. Additionally, we added four items from the Patient-Reported Outcomes Measurement Information System Short Form version 1.0 (PROMIS SF v1.0)–Anxiety 4a and seven items from the Short Screening Scale version of the Diagnostic and Statistical Manual of Mental Disorders, fourth edition (DSM-IV) post-traumatic stress disorder (PTSD) test to screen for anxiety and PTSD.^36,37^

Patients at a participating site were approached consecutively by the research team who screened them for eligibility. Patients who consented to participate were assigned a participant identity (ID), and their anonymized data were entered into a study-specific electronic data capture system using an electronic tablet. Multivariable linear regression analysis was performed to determine associations between patients’ perceived effectiveness of cannabis as the dependent variable and patient and injury characteristics, previous surgery for the pain, pain frequency and severity, and past experience with cannabis as independent variables.

The multivariable regression analysis directed the calculation of the sample size. A significance level of 0.05 was used (conservative estimated effect size of 0.06, powered for 80% and eight predictors), estimating that a sample of 257 patients would provide adequate power to assess the relationship with perceived effectiveness. Therefore, the study aimed to recruit at least 367 patients (presuming 30% incomplete data). The patient recruitment intentionally exceeded the a priori analysis to ensure a well-powered study and enhance the reliability of the statistical analyses.

## Results

### Patient and injury characteristics

The study included 406 patients, with a mean age of 58 (range 20–91) years; 250 (61.6%) were women and 156 (38.4%) were men. Most patients were college-educated (167/404, 41.3%) and had an annual income between CAD 25,000 and 75,000 (205/383, 53.5%). The average reported pain severity score, measured using the VAS, was 55.0 ± 22.7 (52.8–5.72). The majority of patients had chronic pain (375/404; 92.8%), described as pain lasting >6 months; 84.2% of them had experienced pain within the last week (342/406), and 238 (58.8%) had undergone surgery for their injury. Details of patient demographics and injury characteristics are presented in Table 1.

**Table 1.** Patient demographics and injury characteristics.

Variables	Number of patients (%)
Total number of patients	406 (100)
Age (N = 405), mean (range)	58.0 (20–91)
Sex (N = 406)	
Female	250 (61.6)
Male	156 (38.4)
Educational level (N = 440)	
Less than high school	18 (4.5%)
High school	121 (30.0)
College/Trade school	167 (41.3)
Undergraduate degree	71 (17.6)
Graduate degree	27 (6.7)
Income (N = 424)	
<CAD 25,000	80 (20.9)
CAD 25,000–49,999	111 (29.0)
CAD 50,000–74,999	94 (24.5)
CAD 74,999–99,999	53 (13.8)
>CAD 99,999	45 (11.7)
Anxiety (N = 157)	
Yes	38 (24.2)
No	119 (75.8)
PTSD (N = 155)	
Yes	40 (25.8)
No	155 (74.2)
Duration of symptoms (N = 404)	
<6 months (acute)	29 (7.2)
≥6 months (chronic)	375 (92.8)
Underwent surgery for musculoskeletal injury (N = 405)	
No	167 (41.2)
Yes	238 (58.8)
Within the last month	48 (20.2)
During the last 1–12 months	75 (31.5)
>1 year ago	108 (45.5)
Unspecified	7 (2.9)
Experienced musculoskeletal pain in the past week (N = 406)	
Yes	342 (84.2)
No	58 (14.3)
Unsure	6 (1.5)
Pain severity based on VAS scores (N = 401), mean (SD)	55.0 (22.7)
No pain (VAS 0–9)	21 (5.2)
Minimal pain (VAS 10–39)	78 (19.5)
Moderate pain (VAS 40–69)	189 (47.1)
Severe pain (VAS 70–99)	113 (28.2)
Extreme pain (VAS 100)	0 (0.0%)

VAS: visual analog scale; PTSD: post-traumatic stress disorder.

### Analgesics use

Nearly two-third of the patients were prescribed analgesics (256/406, 63.1%); 64.5% (165/256) of them were prescribed opioids and 30.5% (78/256) had used opioids during the last week. Most patients reported oxycodone/oxycontin (64/256, 25.0%) as the most common opioid medication they used and fentanyl as the least common (2/256, 0.8%) (Table 2). Among nonopioid prescription analgesics, naproxen was most commonly used (62/256, 24.2%). Additionally, 315 (78.0%) patients stated that they used nonprescription medications for their arthritis or joint pain, with acetaminophen (208/315, 66.0%) and ibuprofen (158/315, 50.2%) being the most common choices (Table 2). More than a quarter of the patients (105/400, 26.3%) disclosed that they had already used cannabis for medical purposes, and 35.8% (143/400) reported using cannabis recreationally. Additionally, 63 (15.8%) patients reported having used cannabis in the last 12 months specifically for pain control.

**Table 2.** Analgesics use.

Variable	Number of patients (%)	
Use of prescription analgesics for current MSK pain (N = 406)		
Yes	256 (63.1)	
No	150 (36.9)	
Prescription medications	Prescribed for MSK pain (N = 256)	Used during the past week
Opioids	165 (64.5)	78 (30.5)
Oxycodone/Oxycontin	64 (38.8)	25 (15.2)
Codeine	60 (36.4)	30 (18.2)
Hydromorphone	56 (33.9)	24 (14.5)
Morphine (oral)	16 (9.7)	6 (3.6)
Fentanyl (oral/patch)	2 (1.2)	0 (0.0)
NSAIDs	130 (50.8)	62 (24.2)
Naproxen	62 (47.7)	25 (19.2)
Celecoxib	38 (29.2)	17 (13.1)
Diclofenac (topical)	16 (12.3)	10 (7.7)
Toradol	16 (12.3)	3 (2.3)
Meloxicam	14 (10.8)	7 (5.4)
Diclofenac (oral)	13 (10.0)	4 (3.1)
Gabapentin/Pregabalin	17 (6.6)	7 (2.7)
Use of nonprescription/OTC analgesics for current MSK pain (N = 404)		
Yes	315 (78.0)	
No	89 (22.0)	
Nonprescription/over-the-counter medications	Used for MSK pain (N = 315)	Used in the past week (N = 315)
NSAIDs		
Ibuprofen	158 (50.2)	69 (21.9)
Naproxen	100 (31.7)	40 (12.7)
Diclofenac (topical)	37 (11.7)	12 (3.8)
Acetylsalicylic acid	18 (5.7)	7 (2.2)
Other		
Acetaminophen	208 (66.0)	119 (37.8)
Miscellaneous^b^	13 (4.1)	10 (3.2)
Used cannabis, or know someone who has	Medically (N = 400)	Recreationally (N = 400)
No	155 (61.3)	120 (70.0)
Yes,	245 (38.8)	280 (30.0)
Used	70 (17.5)	68 (17.0)
Used and know someone	35 (8.75)	75 (18.6)
Know someone	140 (35.0)	137 (34.6)
Used cannabis in the past 12 months to control pain (N = 399)		
No	336 (84.2)	
Yes	63 (15.8)	

N: number; No: number; MSK: musculoskeletal; OTC: over-the-counter; NSAIDs: nonsteroidal anti-inflammatory drugs.

a Where data were missing or a study participant did not respond to a query, percentages were calculated considering the total number of responses, not the total number of study participants.

b Muscle relaxants, balms/rubs/cream, herbal medications (e.g. arnica and turmeric).

### Perceived effects of cannabis on MSK pain

Overall, patients believed that cannabis was, or could be, used to treat over 50% of their pain (average reduction in VAS pain score = 53.6 ± 2.6, 95% (confidence interval) CI = 51.1%–56.1%) and replace nearly half of their analgesic medications (50.4 ± 3.2, 95% CI = 47.2%–53.6%) (Table 3). Most patients felt comfortable discussing cannabis use with their physicians (77.1 ± 2.7, 95% CI = 74.4–79.8). Our regression analysis showed that several factors were associated with greater reported pain relief with cannabis use. Individuals who had used cannabis for medical purposes (OR = 7.2, 95% CI = 1.6–12.8, *p* = 0.001) and individuals reporting more severe baseline pain (β = 0.2 per point, 95% CI = 0.1–0.3, *p* = 0.012) were more likely to report meaningful pain improvement (Table 4).

**Table 3.** Perceptions regarding cannabis use following MSK injury.

Variable (number of patients)	Mean value (95% CI)
Perceived pain relief/resolution with cannabis use (0% = none, 100% = all) (N = 382)	53.6 (51.1–56.1)
Percentage of their pain medication regimen is made up by cannabis (0% = none, 100% = all) (N = 63)	38.8 (30.4–47.2)
Percentage of analgesic medications that cannabis does/could replace (0% = none, 100% = all) (N = 319)	50.4 (47.2–53.6)
Comfort in discussing cannabis use with provider (0% = not comfortable at all, 100% = completely comfortable) (N = 387)	77.1 (74.4–79.8)

MSK: musculoskeletal; CI: confidence interval.

**Table 4.** Multivariable regression model for patients’ perception of their pain treated by cannabis.

Covariate	OR/ß coefficient	95% CI	p-value
Age	−0.5	−0.3 to 0.1	0.341
Sex			
Male	0.6	−4.7 to 5.9	0.824
Female	−	−	−
Duration of pain/symptoms			
≥6 months (chronic)	3.5	−6.6 to 13.6	0.492
<6 months (acute)	−	−	−
Had surgery	−3.3	−8.6 to 2.0	0.227
Pain severity (VAS)	0.2	0.1 to 0.3	0.001
Used cannabis to manage pain in last year	7.2	1.6 to 12.8	0.012

*p*-value <0.05

VAS: visual analog scale; OR: odds ratio; CI: confidence interval.

Statistics are based on cases with no missing values for any variable used (N = 375).

### Knowledge, attitudes, and preferences regarding cannabis

Only 15.6% of patients (61/391) reported discussing cannabis use with their physicians (Table 5). The number one reason identified by patients for not discussing cannabis as a treatment choice was that they never considered using cannabis for medical purposes (172/330, 52.1%). Concerns about the adverse effects of cannabis and addiction were expressed by 16.4% (54/330) and 11.5% (38/330) of patients, respectively. When asked how they would prefer to obtain cannabis, private dispensaries (26/406, 6.4%) were the first choice, while pharmacies ranked second (23/406, 5.7%). Patients expressed more interest in oral formulations (pills (179/406, 44.1%) and edibles (99/406, 24.4%)) and topical creams (101/406, 24.9%). Inhaled formulations, such as that which could be smoked (69/406, 17.0%) and vaporized preparations (46/406, 11.3%), were less commonly preferred. Regarding the legalization of the recreational use of cannabis in Canada, half of the patients (194/382, 50.8%) either somewhat or strongly supported the legislation.

**Table 5.** Barriers and considerations for clinical use and further investigation.

Variable	Number of patients (%)
Have discussed medical use of cannabis with physician (N = 391)	
No	330 (84.4)
Yes	61 (15.6)
I never thought about using cannabis for medical purposes^a^	172 (52.1)
I don’t need any more medications for pain control^a^	62 (18.8)
I am concerned about adverse effects^a^	54 (16.4)
I am concerned about addiction with cannabis^a^	38 (11.5)
I do not know how to access cannabis^a^	26 (7.9)
I have a moral or religious objection to using cannabis^a^	25 (7.6)
I can easily obtain cannabis through other physicians/sources^a^	18 (5.5)
I don’t think it works^a^	17 (5.2)
My physician doesn’t think it works^a^	15 (4.5)
I prefer to use cannabis without discussing it with my physician^a^	4 (1.2)
Other^a^	39 (11.8)
Was the discussion a positive experience (N = 61)	
Very positive	24 (39.3)
Positive	14 (23.0)
Mixed	17 (27.9)
Somewhat negative	3 (4.9)
Very negative	3 (4.9)
Where patients obtained/preferred to obtain cannabis from	Preferred (N = 406)
Government	40 (9.9)
Private dispensary	26 (6.4)
Pharmacy	23 (5.7)
Online	23 (5.7)
Home grown	19 (4.7)
Forms of cannabis patients preferred to use to treat their pain (N = 406)	
Oral pill/tablet	179 (44.1)
Topical	101 (24.9)
Edible	99 (24.4)
Sublingual	75 (18.5)
Inhaled smoke	69 (17.0)
Inhaled vapor	46 (11.3)
Liquid	40 (9.9)
Transdermal	38 (9.4)
Intra-articular	17 (4.2)
Willingness to participate in a randomized clinical trial comparing cannabis to usual care for pain relief following an MSK injury (N = 381)	
Yes	208 (54.6)
No	80 (21.0)
Unsure	93 (24.4)

MSK: musculoskeletal.

a Denominator is 330 as 330 patients did not discuss cannabis with their providers.

**Figure 1.**
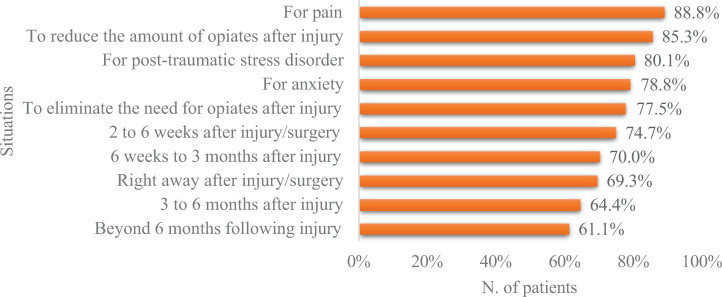
Medical situations and conditions in which patients believed cannabis to be useful for treatment.

Patients identified the initial 3-month period after surgery/injury as the most appropriate period to use cannabis. However, the majority thought that using cannabis at multiple time points could also be beneficial (from immediately after the surgery/injury to beyond 6 months). Furthermore, patients also believed that cannabis may be useful for treating pain (135/152, 88.8%), reducing (128/150, 85.3%) or eliminating the need for opioids (117/151, 77.5%), and treating symptoms of PTSD (121/151, 80.1%) and anxiety (119/151, 78.8%) (Figure 1). More than half of the patients (208/381, 54.6%) expressed their willingness to participate in a randomized clinical trial comparing cannabis to standard treatments for MSK pain (Table 5).

## Discussion

In this study, we investigated the perceptions and views of 406 arthritis patients attending orthopedic and rehabilitation clinics regarding the effectiveness of cannabis use in alleviating joint or arthritis pain. The patients in our study believed that cannabis could relieve 53.6% of their pain and replace at least half (50.4%) of their current pain medications. Furthermore, 26.3% of patients already used cannabis for medical purposes, and 15.8% had used cannabis during the last year, specifically for their joint pain. The other studies conducted at our center examining cannabis perceptions involving patients with a traumatic injury or back pain also support these findings.^33,34^ Studies on chronic and postoperative pain have shown that cannabis and cannabinoid products offer modest but measurable reductions in pain and may exert opioid-sparing effects. This aligns with the perceptions of the patients in the present study.^38–40^

Moreover, our study revealed that 64.5% of pain prescriptions were for opioids, with oxycodone or oxycontin being the most frequently prescribed medications (25.0%). This aligns with findings from other studies, which suggest that, despite efforts to curb opioid prescriptions, opioids remain a common choice for managing chronic MSK pain, including arthritis.^41–44^ Eighty percent of respondents in the study believed that cannabis might lower their reliance on opioids or eliminate the need altogether. This is consistent with results from other studies, according to which, patients who use cannabis for medical reasons reported better pain relief and reported reduced reliance on opioids.^45,46^ In one study, 95% of patients who used cannabis for medical purposes reported improved pain control, and 87% reported using fewer analgesics, including opioids.^47^ Additionally, qualitative research has shown that for individuals with MSK pain (21% arthritis), cannabis is preferred over opioids and considered as the medium for reducing opioid consumption.^48^ These findings strongly suggest that cannabis play an important role in managing MSK joint pain.

With respect to our secondary goals, the most preferred methods of distribution and administration were private dispensaries and oral cannabis formulations, respectively. Although some patients expressed concerns about potential adverse effects and addiction, the primary reason for not discussing cannabis use with their healthcare providers was that they did not consider it as a treatment option for managing their pain. This could be owing to the limited existing literature on the use of cannabis for joint or arthritis-related pain. However, this gap in research highlights the need for high-quality studies, such as RCTs, comparing cannabis to standard pain management treatments for conditions such as arthritis. Currently, cannabis are approved for use in medical conditions such as neuropathic pain, palliative care, chemotherapy-induced nausea, and spasticity associated with multiple sclerosis or spinal cord injury.^49^ Our study indicates that patients are eager to participate in such trials, with over half (54.6%) of our participants expressing an interest in being randomized to either the cannabis or traditional pain management groups in a clinical trial.

Recall bias was a limitation of this study, considering that a survey was used for data collection. Additionally, because cannabis use has historically been associated with negative perceptions, such as concerns over addiction and its legality, concerns regarding social desirability may have also influenced the results. Nevertheless, the high response rate, focus on recent cannabis usage, and the fact that only 7% of participants cited moral or religious objections to cannabis use suggest that these issues had minimal impact on the findings. Moreover, we worked closely with a multidisciplinary team of experts and patients to develop the survey, ensuring that it was designed to meet the study’s objectives. As the study was conducted exclusively in Canada, the generalizability of the findings and methodology to other jurisdictions could be limited. Nevertheless, the timing of the study coincided with a major regulatory moment for cannabis in Canada when its recreational use was legalized, allowing a unique research opportunity. However, it is noteworthy that the dataset were generated in 2018, and perceptions and usage patterns may have changed since then.

With a focus on orthopedic surgery, based on these findings, orthopedic surgeons should proactively address cannabis use and perceptions as part of routine arthritis care. With one section of patients already using cannabis for medical purposes and some viewing it as a potential analgesic, informed discussions may support safer, multimodal pain management strategies. Additionally, educating orthopedic surgeons on appropriate formulations, administration routes, and adverse effects may improve clinical oversight and patient safety and present an opportunity for orthopedic practices to contribute to high-quality research aimed at defining the role of cannabis in evidence-based arthritis management.

## Conclusions

Cannabis use among patients with arthritis and MSK joint pain is highly prevalent, and patients are generally open to discussing it with their healthcare providers. Many patients believe that cannabis is an effective pain management option, particularly during the postoperative recovery phase. Participants also believed that cannabis could address at least 50% of their pain and potentially reduce their reliance on opioids. Data on patient preferences, including preferred methods of administration, distribution, and perceived barriers, can help clinicians and researchers better understand the future use of cannabis for pain management. More research, particularly through RCTs, is essential to assess the safety and efficacy of cannabis use before it can be officially endorsed as a treatment option for this patient group.

## Data Availability

The data supporting the findings of this study are available from the corresponding author upon reasonable request.
